# Targeting Microglial Transcriptional Reprogramming as a Therapy Strategy for Alzheimer's Disease

**DOI:** 10.1002/bies.70186

**Published:** 2026-09-29

**Authors:** Byungwook Kim, Selena S. Wang, Justin R. Kim, Jungsu Kim

**Affiliations:** ^1^ Department of Medical & Molecular Genetics Indiana University School of Medicine Indianapolis Indiana USA; ^2^ Stark Neuroscience Research Institute Indiana University School of Medicine Indianapolis Indiana USA; ^3^ Medical Neuroscience Graduate Program Indiana University School of Medicine Indianapolis Indiana USA

**Keywords:** alzheimer's disease, amyloid, irf8, mef2c, microglia, neuroinflammation, phagocytosis, pu.1 transcription factor, spi1, transcription factor

## Abstract

Recent human genetic studies revealed that Alzheimer's disease risk loci are enriched in genes expressed in myeloid cells, including microglia, the brain's resident immune cells. Notably, several risk‐associated genes, such as *SPI1* and *MEF2C*, encode transcription factors (TFs) that control critical microglial functions, including phagocytosis, inflammatory responses, and neurotrophic support.

The objective of this article is to review the recent progress in understanding these TFs and evaluate them as therapeutic targets rather than as markers of microglial state. We examine the genetic evidence implicating PU.1, MEF2C, IRF8, and BHLHE40/41, the in vivo evidence from animal models, the transcriptional network these factors regulate, and the barriers to clinical translation.

Because these TFs orchestrate broad gene expression networks by regulating multiple disease‐relevant downstream partners, targeting their expression level or activity could achieve greater therapeutic efficacy than targeting single genes, provided that the dose and disease stage of modulation are carefully considered.

## Graphical abstract

Recent human genetic studies highlight the critical role of microglial pathways in Alzheimer's disease, with risk loci enriched in microglia‐specific genes such as SPI1 and MEF2C. Dysregulation of these transcription factors contributes to disease pathogenesis. Targeting them represents a promising therapeutic strategy, as it could simultaneously modulate multiple disease‐relevant pathways.

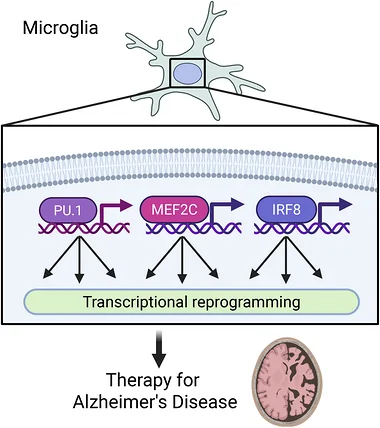

## 1 Introduction

Alzheimer's disease is pathologically characterized by the presence of extracellular amyloid beta (Aβ) plaques and intracellular neurofibrillary tangles (NFTs) in the brain. Aβ is a peptide derived from the transmembrane protein amyloid precursor protein (APP). APP undergoes sequential cleavage by β‐secretase and γ‐secretase, generating Aβ fragments, mainly Aβ40 and Aβ42. The aggregation of Aβ peptides leads to the formation of Aβ plaques [1]. Separately, NFTs are composed of hyperphosphorylated tau protein. While tau normally stabilizes microtubules in neurons, its hyperphosphorylation leads to misfolding and aggregation into paired helical filaments (PHFs), resulting in the accumulation of NFTs [2]. These pathological features are accompanied by neuronal and synaptic loss, chronic inflammation, and oxidative stress. The accumulation of Aβ plaques and NFTs is hypothesized to lead to neurodegeneration, causing cognitive impairment and behavioral deficits [3, 4].

Recent human genetic studies of Alzheimer's disease have highlighted the role of the immune system and neuroinflammation in the onset of disease by identifying genetic variants associated with Alzheimer's disease, particularly those expressed in microglia [5, 6, 7]. Microglia, the resident immune cells of the brain, play dual roles in Alzheimer's disease pathogenesis. Under normal conditions, they help maintain homeostasis by providing neurotrophic support and clearing harmful substances, such as amyloid plaques, through phagocytosis [8, 9, 10]. However, when microglia become dysfunctional, they can release inflammatory factors that damage neurons and synapses, ultimately contributing to disease pathogenesis [11]. This imbalance between protective and detrimental microglial functions is a key factor in Alzheimer's disease pathology.

Transcription factors play crucial roles in regulating gene expression, a fundamental process essential for all cellular functions [12]. By orchestrating gene expression, transcription factors influence whether microglia play protective or detrimental roles in maintaining brain homeostasis and responding to disease [8, 13]. Therefore, modulating the level or activity of transcription factors represents a promising therapeutic strategy for developing effective treatments for complex diseases, such as Alzheimer's disease. Transcription factors by nature modulate the expression of multiple downstream targets in intricately coordinated and co‐regulated pathways [12, 14]. Given the multifactorial etiology of Alzheimer's disease, targeting transcription factors offers a unique opportunity to simultaneously modulate multiple disease‐related pathways. Consequently, when identifying potential therapeutic targets, it is important to move beyond a reductionist perspective that focuses on a single protein. Instead, we need to adopt strategies that address multifactorial disease pathogenesis. Despite the significant therapeutic potential of targeting transcription factors, their effective implementation faces considerable challenges. These include the inherent complexity of their protein‒protein interactions [15] and the lack of readily druggable pockets for most transcription factors [16]. However, recent advancements in drug discovery, such as the development of novel chemical libraries and protein engineering techniques, are paving the way for overcoming these obstacles [15, 17, 18].

This review explores the role of microglial transcription factors in Alzheimer's disease pathogenesis, highlighting recently identified genetic risk factors. Furthermore, to define microglia‐specific transcriptional networks centered on PU.1, MEF2C, and IRF8, we explore the potential of these transcription factors functioning as therapeutic targets for Alzheimer's disease treatment by integrating genetics, epigenomics, and functional evidence. We hypothesize that targeted transcriptional reprogramming of microglia, achieved by modulation of core microglial transcriptional factors such as PU.1, MEF2C, and IRF8, can shift microglia from a disease‐promoting state toward a state that enhances amyloid and tau clearance, reduces maladaptive inflammation, and preserves synaptic integrity. Because these transcriptional factors coordinate broad gene programs, selective modulation of them may produce better therapeutic effects compared with other single‐gene interventions. We further suggest that combinatorial or temporally tuned modulation of multiple transcriptional factors may allow fine control of microglial function with improved efficacy and reduced adverse consequences.

## 2 Microglial Transcription Factors Associated with Alzheimer's Disease

Human genetic studies of Alzheimer's disease have significantly advanced our understanding of the disease's etiology, particularly through the identification of new genetic variants associated with Alzheimer's disease [19]. Interestingly, some Alzheimer's disease risk loci are highly enriched within or near genes encoding microglia‐specific transcription factors, highlighting their crucial roles in the pathogenesis of Alzheimer's disease (Table 1) [5, 7, 20, 21].

**TABLE 1:** Microglial transcription factors identified from Alzheimer's disease genetics studies.

TF	SNP ID	Chr	SNV	Association with AD	References
PU.1 (*SPI1*)	rs1057233	11	G>A	Associated with the reduced risk of developing Alzheimer's disease	[22]
	rs3740688	11	G>A/G>T	Associated with the reduced risk of developing Alzheimer's disease	[23]
	rs78245530	11	G>T	Associated with the reduced risk of developing Alzheimer's disease	[24]
MEF2C	rs190982	5	G>A,C	Associated with the reduced risk of Alzheimer's disease	[25, 26]
	rs9293506	5	C>A,G,T	Associated with the increased risk of cognitive aging	[27]

Abbreviations: Chr, chromosome; SNP, Single‐nucleotide polymorphism; SNV, single‐nucleotide variant; TF, Transcription factor.

*SPI1* gene, encoding the microglial transcription factor PU.1 was reported as a genetic risk factor for Alzheimer's disease [22]. A single‐nucleotide polymorphism (SNP) rs1057233, located near the *SPI1* gene, is associated with a reduced risk of developing Alzheimer's disease. This protective effect is associated with lower *SPI1* expression in myeloid cells, such as monocytes and macrophages [22]. Furthermore, the SNPs rs3740688 and rs78245530 are significantly associated with Alzheimer's disease in European [23] and Chinese [24] populations, respectively. Interestingly, these variants are also suggested to reduce the risk of developing Alzheimer's disease in these specific populations [23, 24]. In addition to *SPI1*, the SNPs rs190982 and rs9293506 within the microglial transcription factor gene Myocyte Enhancer Factor 2C (MEF2C) have been linked to Alzheimer's disease risk and cognitive aging in Caucasian [25] and Taiwanese [27] populations, respectively.

These microglial transcription factors, associated with Alzheimer's disease risk, regulate various microglial functions, including immune responses (such as phagocytosis, cytokine production, and neuroinflammation) and lipid metabolism. Therefore, new insights learned from studying these transcription factors are crucial for developing targeted therapies and improving our understanding of Alzheimer's disease pathogenesis.

## 3 SPI1/Pu.1

### 3.1 Introduction of SPI1/PU.1

PU.1 is a transcription factor essential for the development and function of myeloid cells, particularly microglia, the resident immune cells of the brain [28, 29]. Microglial development originates in primitive myeloid progenitors and depends on the transcription factors PU.1 and interferon regulatory factor (IRF) 8 [29]. PU.1 and IRF8 cooperate to exert synergistic effects through a complex IRF‐Erythroblast Transformation Specific (ETS) motif. PU.1, as an ETS family transcription factor, binds specific DNA sequences known as ETS motifs, which are located predominantly in the enhancers but also in the promoters of target genes [30, 31]. It functions both intrinsically and through the regulation of its target genes *Irf8*, *colony stimulating Factor 1 (Csf1), and Csf1 receptor (Csf1r)* [32], which are themselves known to be critical for microglial survival and proliferation [33, 34, 35]. In addition, PU.1 influences immune responses by regulating the expression of *allograft inflammatory factor 1* (*Aif1, also known as ionized calcium‐binding adapter molecule 1, Iba1), C‐X3‐C motif chemokine receptor 1 (Cx3cr1), triggering receptor expressed on myeloid cells 2 (Trem2)*, and *TYRO protein tyrosine kinase binding protein (Tyrobp)* [32, 36]. Previous in vitro studies have also demonstrated that PU.1 regulates Alzheimer's disease‐associated genes involved in the immune response and phagocytic activity in microglia, including *C‐C motif chemokine ligand* (*Ccl2), C‐X‐C motif chemokine ligand 2 (Cxcl2), Aif1, Ms4a4a, apolipoprotein E (ApoE), clusterin (Clu, also known as ApoJ), Lyn, cathepsin B (Ctsb), and hexosaminidase subunit beta (Hexb)* [22, 37, 38, 39].

In summary, PU.1 is essential for the development of microglia from primitive myeloid progenitors and enables microglia to respond to pathogens, cellular debris, and neurodegenerative signals. It functions by binding to enhancer and promoter regions of target genes, thereby regulating the expression of genes involved in the immune response and phagocytosis processes. Because immune pathways are critical for Alzheimer's disease pathogenesis, remodeling of the transcriptome by PU.1 may affect the neuropathological features of Alzheimer's disease.

### 3.2 Targeting PU.1 as a Therapeutic Strategy for Alzheimer's Disease

Previous studies investigating the therapeutic potential of PU.1 for Alzheimer's disease have relied primarily on in vitro experiments using cell culture systems [22, 37, 38]. However, these cell‐based models have inherent limitations in accurately replicating the intricate complexity of the brain and the dynamic interactions among its diverse cell types. In addition, it remains uncertain whether the knockdown or overexpression of PU.1 would be effective in treating Alzheimer's disease because of two conflicting hypotheses. On the one hand, the knockdown of PU.1 in BV‐2 microglia stimulated with LPS reduces the expression of proinflammatory genes and immune response mediators, whereas the overexpression of PU.1 has the opposite effect under the same conditions [22, 37]. On the other hand, PU.1 positively regulates phagocytosis, a potentially beneficial process critical for Aβ clearance in Alzheimer's disease pathology [37, 38]. This creates a contradiction in determining whether PU.1 inhibition or activation would be the more appropriate therapeutically. Consequently, the net effect of modulating PU.1 levels in vivo on Alzheimer's disease pathology, and therefore, the optimal therapeutic strategy, remains unclear.

To address this knowledge gap, we recently performed a study using PU.1‐knockdown and PU.1‐overexpression mouse models crossbred with amyloidosis mouse models [40]. Our recent results demonstrated that PU.1 knockdown exacerbated the pathological features of Alzheimer's disease, including elevated Aβ peptide levels and increased amyloid plaque burden, in 4 month old mice. Conversely, PU.1 overexpression ameliorated these features as well as dystrophic neurites in mice of the same age [40]. In contrast to a previous in vitro study reporting that knockdown of PU.1 reduces neuroinflammation [37], our findings in amyloidosis mouse models revealed that PU.1 knockdown increased gliosis, whereas PU.1 overexpression decreased it [40]. These findings suggest that the effects of PU.1 on Aβ clearance, mediated by phagocytosis, also modulate the immune response in Alzheimer's disease.

Furthermore, a previous study explored the mechanistic link between PU.1 and interleukin‐33 (IL‐33) [41]. IL‐33 injection enhanced PU.1 transcriptional activity in an amyloid mouse model, resulting in increased Aβ clearance. Notably, this beneficial effect was abolished by intracerebroventricular administration of the pharmacological PU.1 inhibitor [41]. In contrast, another recent study reported that oral administration of a functional PU.1 inhibitor for six weeks reduceed amyloid plaque deposition in a 1‐year‐old amyloid mouse model and decreases tau phosphorylation in a 1‐year‐old tauopathy mouse model [42]. These seemingly conflicting studies highlight the need for further investigation into PU.1 modulation, with careful consideration of critical variables such as treatment duration, drug formulation, and the specific stage of Alzheimer's disease pathology being targeted.

## 4 MEF2C

### 4.1 Introduction of MEF2C

MEF2C is a transcription factor with crucial roles in immune regulation, synaptic plasticity, and neuronal survival [43, 44]. MEF2C exerts anti‐inflammatory effects within the brain by suppressing the expression of proinflammatory cytokines and protecting neurons from inflammatory damage. Notably, MEF2C expression is reduced in an interferon‐type Ι (IFN‐Ι)‐dependent manner during brain aging [45]. In a 5xFAD mouse model of Alzheimer's disease, decreased microglial MEF2C nuclear translocation contributes to chronic neuroinflammation, which exacerbates neurodegeneration [46].

In addition, recent research in a mouse model of tauopathy revealed the tau‐driven neuroinflammatory axis in microglia. In this axis, pathological tau activates the cyclic GMP‐AMP synthase (cGAS)‐stimulator of interferon genes (STING)‐IFN‐Ι pathway in microglia, leading to the suppression of MEF2C, a critical transcription factor for cognitive resilience [47].

### 4.2 Targeting MEF2C as a Therapeutic Strategy for Alzheimer's Disease

Previous studies have shown that reduced neuronal MEF2C levels, due to neuroinflammation or aging, impair synaptic maintenance, subsequently leading to increased synapse loss [45, 46]. A recent study further demonstrated that AAV‐induced Mef2c knockdown increased Aβ deposition, triggered neuronal apoptosis, reduced levels of the synaptic proteins, postsynaptic density protein 95 (PSD95) and synaptophysin (SYP), and impaired learning and memory abilities in an amyloid mouse model [48]. Conversely, AAV‐induced overexpression of neuronal Mef2a/c improved cognitive function in the fear conditioning test and reduced hyperexcitability in a tauopathy mouse model [49].

While previous research on the therapeutic potential of MEF2C has primarily focused on its role in neuronal cell types, future research needs to prioritize microglia‐specific functions to better define the diverse roles of MEF2C across different Alzheimer's disease contexts.

## 5 Other Microglial Transcription Factors Associated with Alzheimer's Disease

### 5.1 Interferon Regulatory Factor 8 (IRF8)

Recent studies have elucidated the critical role of IRF8 in Alzheimer's disease pathogenesis through its regulation of neuroinflammation, microglial activation, and synaptic pruning. IRF8 is a transcription factor that cooperates with PU.1 to regulate the development and function of immune cells, particularly microglia [29]. Furthermore, PU.1 and IRF8 activate each other through a positive feedback loop [29]. PU.1 directly induces IRF8 transcription by binding to its enhancer and promoter [50], while IRF8 reciprocally enhances PU.1 expression by targeting an upstream regulatory element of the PU.1 gene [29, 39]. They bind to composite IRF‐ETS motifs, driving the expression of proinflammatory genes, such as IL‐1β and tumor necrosis factor‐alpha (TNF‐α), during microglial activation [39].

Notably, *Irf8* deletion in the microglia did not alter overall Aβ accumulation in both 4‐ or 12‐month‐old 5xFAD female mice. However, in 12‐month‐old female mice, microglial *Irf8* deletion reduced plaque size while increasing the number of plaques [35]. This effect was probably due to impaired phagocytic function, as *Irf8* deletion significantly decreased the number of Methoxy‐X04 (fluorescent Aβ probe)‐positive microglia in 6‐month‐old male mice in an in vivo phagocytosis assay [35]. To clarify these somewhat complex phenotypes, it is warranted to use age‐matched and same sex mice to determine plaque levels and to perform an in vivo phagocytosis assay.

### 5.2 Basic Helix‐Loop‐Helix Family, Members e40 and e42 (BHLHE40 and BHLHE41, Collectively Termed BHLHE40/41)

The transcription factors BHLHE40/41 play roles in immune cell differentiation and function. They are involved in regulating the activity of macrophages, T cells, and other immune cells [51]. BHLHE40, in particular, is known to suppress proinflammatory cytokine production, helping to control immune responses [52].

The transcription factors BHLHE40/41 are emerging as important players in Alzheimer's disease pathology, primarily through their regulation of disease‐associated microglia/lipid‐associated macrophage (DAM/LAM) responses. Specifically, BHLHE40/41 regulate genes involved in lipid clearance (ABCA1, APOE, LPL), lysosomal processing (CTSD, PSAP, LAMP‐ 1 and ‐2), TREM2, metabolism (PPARγ, LXR), and efferocytosis (the process by which apoptotic cells are cleared by phagocytic cells) [53]. Furthermore, a recent study shown that the transcription factor EB (TFEB), a key regulator of lysosomal and autophagy, is negatively regulated by BHLHE40/41 [54]. This regulatory loop, mediated by BHLHE40/41, could improve our understanding of lysosomal dysfunction in Alzheimer's disease.

While direct links between BHLHE family proteins and Aβ or tau pathology have not yet been well established, their roles in inflammation and microglial function suggest potential interactions with Alzheimer's disease‐related pathways. Therefore, further research is still warranted to establish a more definitive understanding of the roles played by BHLHE40/41 in Alzheimer's disease.

## 6 Common Regulatory Networks Regulated by PU.1, MEF2C, IRF8, and BHLHE40/41

To broaden the scope of potential drug targets for neurodegenerative diseases, including Alzheimer's disease and other diseases with strong involvement of neuroinflammation, we summarized transcriptional targets regulated by PU.1, MEF2C, IRF8, and BHLHE40/41. As shown in Figure 1, previous studies showed that PU.1 induces the transcription of several surface receptors, such as Integrin subunit alpha M (ITGAM, also known as CD11b), vascular cell adhesion molecule 1 (VCAM1, also known as CD106), TREM2, and TYROBP [32, 55], thereby enhancing microglial phagocytic capacity for Aβ clearance [56, 57]. Additionally, IL‐33 requires PU.1 activity to enhance microglial phagocytic function to Aβ clearance [41]. Moreover, a recent study reported that IL‐33 indirectly induces VCAM1 expression in microglia, enhancing microglial chemotaxis toward Aβ plaques and facilitating Aβ clearance in a mouse model of amyloidosis [57]. PU.1 also functions as an immune activator by inducing the expression of IL‐9 and CD80 [58, 59, 60].

**FIGURE 1:**
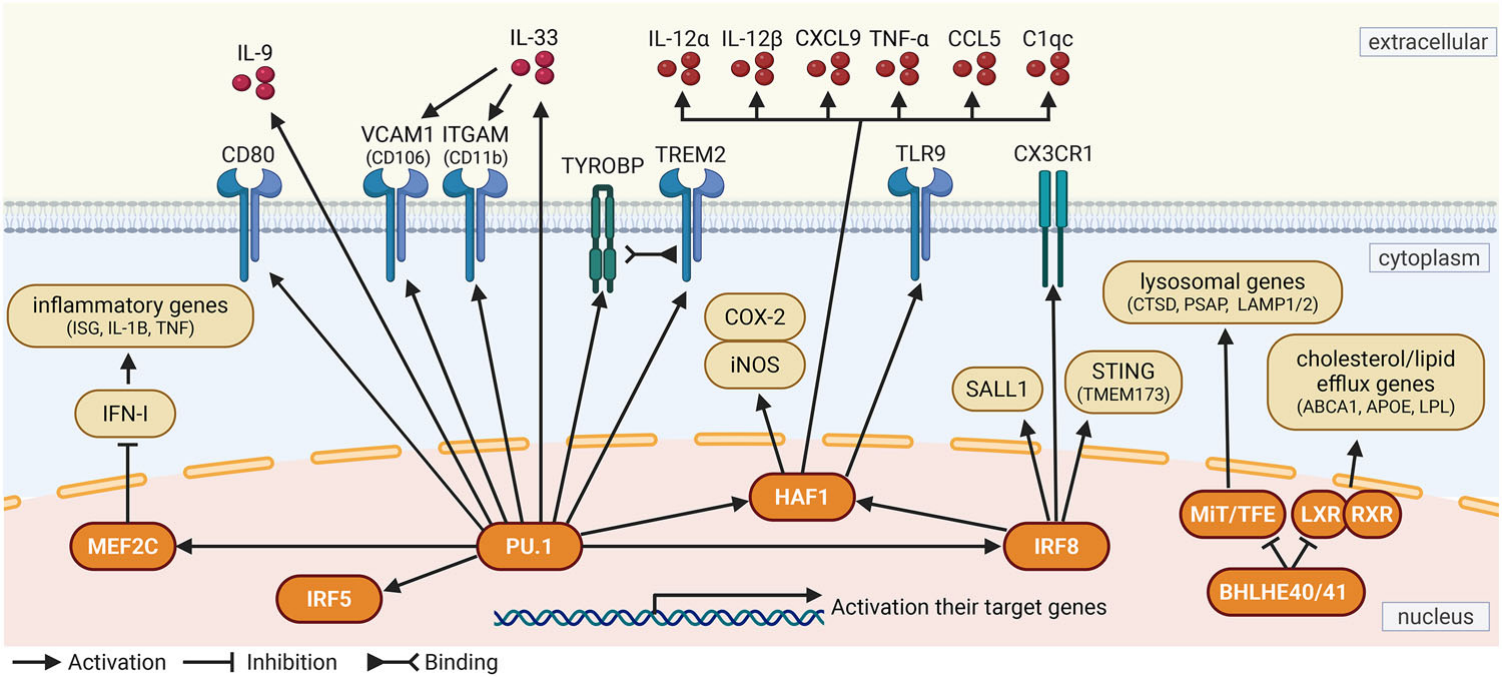
Microglial gene regulatory networks regulated by PU.1, MEF2C, IRF8, and BHLHE40/41. Transcriptional factors, PU.1, MEF2C, IRF8, and BHLHE40/41 play pivotal roles in regulating the expression of various immune‐related genes. i) PU.1 directly regulates the expression of many surface receptors and transcription factors, including VCAM1, ITGAM, TYROBP, TREM2, CD80, MEF2C, and IRF8 [32, 55]. This regulatory network contributes to the balance of immune surveillance, phagocytosis, and inflammation [32, 55, 56, 57]. ii) PU.1 collaborates with its downstream partners (IRF8, HAF1, MEF2C) to induce the expression of cytokines and chemokines (IL‐9, IL‐12α, IL‐12β, IL‐33, CXCL9, TNF‐α, and CCL5), complement component (C1qc), pro‐inflammatory enzymes (COX‐2 and iNOS), and surface receptor TRL9 [41, 58, 59, 60, 61, 62]. iii) IRF8, directly activated by PU.1, further contributes to the activation of downstream targets including SALL1, CX3CR1, and STING (TMEM173), which collectively play key roles in chemokine signaling, and innate immune response, including type I interferon (IFN‐I) production [35, 63]. MEF2C, another downstream target of PU.1 [64], helps sustain the homeostatic and surveillant state of microglia by suppressing pro‐inflammatory genes, specifically through the inhibition of the IFN‐I response [45]. iv) BHLHE40/41 regulate lipid metabolism and lysosomal degradation by repressing the transcriptional responses induced by LXR:RXR nuclear receptors and MiT/TFE family transcription factors (such as TFEB and MITF), forming a negative feedback loop [53, 54]. The solid pointed arrows between nodes indicate activation, while the T‐shaped arrows indicate inhibition. The Y‐shaped arrows represent binding interactions.

The transcription factor hematopoiesis associated factor 1 complex (HAF1) is known to form dimeric or trimeric configurations with the transcription factors, PU.1, IRF1, and IRF8 [61, 62]. HAF1 and PU.1 are both functionally and physically connected, with PU.1 serving as a core component of the HAF1 complex [65]. The HAF1 complex activates a range of genes associated with microglial activation, including *inducible nitric oxide synthase* (*iNOS), Cyclooxygenase‐2 (COX‐2), TNF‐α, IL‐12α, IL‐12β, CXCL9, CCL5*, and complement components such as the *complement C1q C chain* (*C1qc*) (Figure 1) [31, 35, 39, 61]. In addition, PU.1‐IRF8 complex functions as an immune activator, and both transcription factors are essential for microglial activation [39]. Moreover, IRF8 is directly activated by PU.1 and contributes to the activation of SALL1, CX3CR1, and STING (also known as TMEM173) [35, 63]. The STING interacts with cGAS and plays a significant role in Alzheimer's disease by promoting microglial dysfunction and neuroinflammation [66, 67]. For example, pharmacological inhibition of STING activation reduces microglia‐mediated inflammatory response and ameliorates a wide range of AD‐related symptoms in App^NL−G−F^/hTau double‐knock‐in mice [68].

Especially, PU.1 functions as a pioneer transcriptional factor that establishes enhancer accessibility in myeloid lineage cells and directly induces expression of other transcriptional factors (e.g., IRF8, MEF2C) [29, 64, 69, 70]. Mechanistically, PU.1 binds ETS motifs and cooperates with IRF family members at composite IRF‐ETS elements to regulate microglial immune programs (phagocytic activity, cytokine production, TLR signaling, and complement activation) [39, 70, 71]. Additionally, PU.1 occupancy at MEF2C regulatory regions has been reported [64], and MEF2C in turn regulates genes involved in synaptic support and anti‐inflammatory responses [45]. IRF8 reciprocally stabilizes PU.1 expression via enhancer interactions, forming a positive feedback loop that can potentiate immune activation [39]. BHLHE40/41 bind to open chromatin at promoters and enhancers of LXR:RXR and MiT/TFE family transcriptional factors, acting as transcriptional repressors and modulating cholesterol/lipid efflux genes and lysosomal genes, thereby influencing cellular clearance functions (Figure 1 and Table 2) [53, 54].

**TABLE 2:** Network and cellular functions of target genes regulated by PU.1, MEF2C, IRF8, and BHLHE40/41.

TF	Network TF	Target (s)	Suggested Function	References
PU.1	IRF8	CSF, CSF1r	microglial survival and proliferation	[32, 33, 34, 35]
PU.1	IRF8 HAF1	CCL5, C1QC, TLR9, CXCL9, TNF‐α, IL‐12	activates immune responses and phagocytic activity in microglia	[31, 35, 39, 61]
PU.1	IRF8	SALL1, CX3CR1, TMEM119	microglial identity and innate immune response	[35, 63]
PU.1	MEF2C	IFN‐I, IL‐1B, TNF	Synaptic support and anti‐inflammatory responses	[45, 46]
BHLHE40/41	MiT/TFE family	CTSD, PSAP, LAMP‐1 and ‐2	negatively regulate lysosomal biogenesis	[53, 54]
BHLHE40/41	LXR;RXR	ABCA1, APOE, LPL	negatively regulate lipid clearance and storage	[53]

Taken together, the microglial transcription factors PU.1, MEF2C, IRF8, and BHLHE40/41 are crucial in various immune system processes, regulating the expression of key pro‐inflammatory mediators, cytokines, and innate immune receptors. This broad regulatory function highlights the significance of PU.1, MEF2C, IRF8, and BHLHE40/41 in immune surveillance, phagocytosis, inflammation, lipid clearance, lysosomal processing, and their potential involvement in autoimmune or neuroimmune conditions (Figure 1).

## 7 Translational Challenges for Transcription Factor Modulators

Recent preclinical studies targeting PU.1, MEF2C, and IRF8 in mouse models have demonstrated significant amelioration of amyloid pathology, neuroinflammation, tau pathology, and cognitive decline (Table 3). However, despite their therapeutic potential, microglial transcription factors have not yet progressed to human clinical trials.

**TABLE 3:** Studies of microglial transcription factors associated with Alzheimer's disease risk in mouse models.

TF	Amyloid or Tau Mouse model	Age (month)	sample size	Sex	Approach	Region	Finding	References
PU.1 (*SPI1*)	APPPS1‐21	4	N = 13‐15/genotype	♂♀	cross with PU.1 knockdown mice	cortex, hippocampus	Aβ levels↑, Amyloid burden↑, microgliosis↑, astrogliosis↑, Aβ phagocytosis↓	[40]
	5xFAD	4	N = 19‐21/genotype	♂♀	cross with PU.1 transgenic mice	cortex, hippocampus	Aβ levels↓, Amyloid burden↓, microgliosis↓, astrogliosis↓, dystrophic neurites↓, Aβ phagocytosis↑	[40]
	APPPS1‐21	4	N = 19‐20/genotype	♂♀	postnatal microglia PU.1 deletion	cortex, hippocampus	Aβ levels↑, Amyloid burden↑, microgliosis↑, astrogliosis↑, Aβ phagocytosis↓, dystrophic neurites↑	[72]
	5xFAD	6	N = 5‐7/genotype	♂♀	postnatal microglia PU.1 deletion	Subiculum	No change in ThioS (+) amyloid burden, but anti‐Aβ antibody (+) amyloid burden↓, lipid droplet in microglia↓	[73]
	APP/PS1 (dE9)	10‐12	N = 7/group	Not specified	PU.1 inhibitor (DB2313) with IL‐33 injection	cortex	IL‐33 treatment = > Aβ clearance ↑ & Amyloid burden ↓, DB2313 abolished these phenotypes	[41]
	5xFAD	12	N = 4‐5/group	♂	PU.1 inhibitor (A11) daily treat for 6‐weeks	Hippocampus	Aβ plaque burden↓	[42]
	PS19 (P301S)	12	N = 7‐10/group	♂	PU.1 inhibitor (A11) daily treat for 6‐weeks	hippocampus	phosphorylated tau↓, learning and memory abilities↑	[42]
MEF2C	APP/PS1 (dE9)	6, 10	N = 10/group	Not specified	AAV‐induced Mef2c knockdown	cortex	Amyloid burden↑, apoptosis↑, PSD95 and SYP proteins↓, learning and memory abilities↓	[48]
	PS19 (P301S)	6	N = 10/group	Not specified	AAV‐induced Mef2c knockdown or overexpression	prefrontal cortex	Mef2a/c overexpression = > cognition↑, hyperexcitability↓	[49]
IRF8	5xFAD	12	N = 5‐8/genotype	♀	postnatal microglia IRF8 deletion	cortex	Aβ plaque sizes ↓, number of plaques↑	[35]
		6	N = 3‐4/genotype	♂	postnatal microglia IRF8 deletion	cortex	interaction of microglia with Aβ plaque↓, microglial phagocytosis activity↓	

Before clinical translation can begin, several barriers need to be addressed. Because these transcription factors are broadly expressed across myeloid lineages, systemic modulation risks disrupting peripheral immunity [74, 75, 76]. Therefore, microglia‐selective targeting strategies are essential to minimize peripheral immune complications [77, 78, 79]. Drug delivery to brain microglia remains a major challenge due to the blood‐brain barrier (BBB) [80]. Therefore, it is important to explore various delivery routes, including oral administration, intracerebral or intrathecal injection, BBB‐penetrant nanoparticles, and ligand‐targeted systems [80, 81]. In addition, potential off‐target effects need to be carefully evaluated, as the modulation of transcription factors can influence unintended other downstream pathways, leading to alteration of metabolism and/or systemic immune responses [74, 82]. Therefore, a comprehensive preclinical safety and peripheral immune assessment is necessary before clinical translation.

Furthermore, it is critical to determine optimal dosage and appropriate disease stage for transcription factor modulation, as these factors often display a nonlinear dose‐response relationship [83]. Even modest changes in transcription factor levels can modulate other transcriptional programs, and supraoptimal dosing may produce adverse outcomes [84, 85]. Consequently, preclinical studies need to include dose‐titration experiments using CRISPRa, CRISPRi, or pharmacological dosage approaches. These experiments should be combined with multiple functional assays, including phagocytic capacity, cytokine production/secretion, microglial morphology, and in vivo cognition test, to establish a therapeutic window for clinical translation. Therapeutic modulation should also be tailored to disease stage. For example, in early‐stage Alzheimer's disease, increasing PU.1 activity may enhance microglial amyloid clearance [38, 40]. However, in the later stages, it might be beneficial to reduce PU.1 activity while increasing MEF2C activity to inhibit harmful inflammation and support neuroprotective microglial functions [22, 37, 42, 46]. This stage‐dependent framework underscores the need to define both optimal dosing and disease‐specific timing before advancing transcription factor‐targeted therapies to clinical evaluation.

## 8 Conclusion

Microglial transcription factors regulate immune responses and maintain brain homeostasis [13]. Dysregulation of these factors significantly contributes to Alzheimer's disease pathogenesis by driving neuroinflammation, impairing the clearance of Aβ and tau, and exacerbating neuronal damage. Furthermore, the regulatory networks of these transcription factors are specific to the distinct microglial phenotypes observed in Alzheimer's disease, including the endolysosomal phenotype and inflammatory effector cluster [86]. Specifically, PU.1, IRF8, and BHLHE40/41 regulates multiple downstream targets that orchestrate microglial immune response functions, including phagocytosis, cytokine production, and inflammatory signaling, while MEF2C functions as a counterbalance by suppressing excessive pro‐inflammatory responses [31, 32, 33, 34, 35, 39, 45, 46, 53, 54, 61, 63]. Therefore, it is essential to thoroughly understand how microglial transcriptional regulation and Alzheimer's disease risk factors interact functionally. This knowledge is crucial for developing targeted therapies that can effectively modulate microglial function and slow disease progression. Current evidence suggests that microglial activation and functional changes, prominently observed in cortical and hippocampal regions during Alzheimer's disease pathology, may be ameliorated through transcriptional remodeling within microglia [35, 40, 41, 42, 48, 49].

However, given that these transcription factors regulate multiple target genes, further investigations utilizing Alzheimer's disease models are essential to fully elucidate and validate these potential mechanisms. Understanding these mechanisms could provide valuable insight into potential therapeutic targets of transcription factors for mitigating microglial dysfunction in Alzheimer's disease.

## Author Contributions

Byungwook Kim and Jungsu Kim conceived the overall contents. Byungwook Kim, Selena S. Wang, and Justin R. Kim drafted the manuscript. Byungwook Kim, Selena Wang, and Jungsu Kim designed and prepared all the graphs and figures. Byungwook Kim and Jungsu Kim edited the manuscript.

## Conflicts of Interest

The authors declare no conflicts of interest.

## Data Availability

Data sharing is not applicable to this article, as no new data were created or analyzed in this study.
